# Risk perception of blood transfusions – a comparison of patients and allied healthcare professionals

**DOI:** 10.1186/s12913-018-2928-x

**Published:** 2018-02-17

**Authors:** Jan A. Graw, Katja Eymann, Felix Kork, Martin Zoremba, Rene Burchard

**Affiliations:** 1 Massachusetts General Hospital, Harvard Medical School, Boston, MA USA; 2 Department of Anesthesiology and Operative Intensive Care Medicine (CCM, CVK), Charité – Universitätsmedizin Berlin, Berlin, Germany; 3 Department of Anesthesiology, Intensive Care Medicine and Emergency Medicine, Kreisklinikum Siegen, Siegen, Germany; 4 Department of Anesthesiology, University Hospital RTWH Aachen, Aachen, Germany; 5 Department of Trauma and Orthopedic Surgery, Kreisklinikum Siegen, Siegen, Germany; 6 Department of Health, University Witten/Herdecke, Kreisklinikum Siegen, Weidenauer Str. 76, 57076 Witten, Germany

**Keywords:** Blood transfusion, Risk perception, Doctor-patient communication, Caregiver

## Abstract

**Background:**

Due to an increasing demand in health care services plans to substitute selective physician-conducted medical activities have become attractive. Because administration of a blood transfusion is a highly standardized procedure, it might be evaluated if obtaining a patient’s consent for a blood transfusion can be delegated to allied healthcare professionals. Physicians and patients perceive risks of transfusions differently. However, it is unknown how allied healthcare professionals perceive risks of transfusion-associated adverse events.

**Methods:**

Patients (*n* = 506) and allied healthcare professionals (*n* = 185) of an academic teaching hospital were asked to quantify their concerns about transfusions including five predefined transfusion-associated risks and their incidences.

**Results:**

Blood transfusions were considered to be generally harmful by 10.9% of patients and 14.6% of caregivers (*P* = 0.180). Among all surveyed patients, 36.8% were worried about infection-transmissions (caregivers: 27.6%; *P* = 0.024). Compared to 5.4% of caregivers, 13.6% of patients believed infection-transmission was a frequent complication (*P* = 0.003). Caregivers ranked the risks of receiving an AB0-mismatch transfusion (caregivers: 29.7% vs. patients: 19.2%, P = 0.003) or a transfusion-associated allergic reaction (caregivers: 17.3% vs. patients: 11.1%, *P* = 0.030) significantly higher than patients and were aware of the high incidence of transfusion-associated fever (caregivers: 17.8% vs. patients: 8.3%, *P* < 0.001).

**Conclusion:**

A significant part of interviewees perceived transfusions as a general health hazard. Patients perceived infection-transmissions as the most frequent and greatest transfusion-associated threat while caregivers focused on fatal AB0-mismatch transfusions and allergic reactions. Understanding the patients’ main concerns about blood transfusions and considering that these concerns might differ from the view of healthcare professionals might improve the process of shared decision making.

**Electronic supplementary material:**

The online version of this article (10.1186/s12913-018-2928-x) contains supplementary material, which is available to authorized users.

## Background

Blood transfusions belong to the most common medical interventions and can be a life saving therapy in medical emergencies. Advanced screening methods, meticulous donor selection, and modern blood processing procedures guarantee the current very high safety standard of allogenic blood transfusions [1, 2].

In the early 1980s, a significant outbreak of HIV infections associated with blood transfusions raised the general public’s awareness for the risk of infection transmissions by allogenic blood transfusions [3]. In addition, there are further adverse effects that can occur with transfusion of allogenic blood products. Noninfectious complications like transfusion associated lung injury (TRALI) and hemolytic transfusion reactions including ABO-mismatch transfusions are responsible for most of the very rare lethal transfusion-associated complications [4]. More frequently but with less severe consequences, blood transfusions are followed by immunologic adverse effects such as allergic or non-hemolytic febrile transfusion reactions [5]. Bacterial contamination of allogenic blood products is another adverse effect that can occur during blood processing and storage.

Based on the increasing demand of healthcare services and concomitant shortage of physicians in recent years, substitution of physician-based medical activities by allied healthcare professionals has gained interest of health care providers and politicians [6, 7]. Administration of a blood transfusion is a highly standardized procedure. The expected benefit of a transfusion needs to be balanced carefully against the potential risks of unwanted side effects. Physicians are trained to evaluate risks, benefits and side effects of their medical therapies and should know risks and incidences of common adverse effects associated with transfusion of allogenic blood products.

Only few data exist on patients’ risk perceptions of blood transfusions. Recently, Vetter and colleagues reported that patients perceive the risk and incidence of infection transmissions associated with blood transfusions significantly higher than anesthesiologists and surgeons [8]. However, it is unclear whether transfusion-associated risk perceptions of non-physician healthcare professionals that are regularly exposed to transfusion procedures but not involved in transfusion-associated decision-making differ from transfusion-associated risk perception of the general public.

In this study we surveyed a cohort of in- and outpatients of an academic teaching hospital about their risk perception and knowledge related to blood transfusions. Furthermore, we compared the results to responses obtained from a group of allied health care professionals in the same institution.

## Methods

The Medical Ethics Committee of the Medical Council Westphalia-Lippe approved this study (number of ethical approval: 2015–424-f-S). Written informed consent was obtained from all study subjects survey before participation via the initial page of the paper survey.

### Setting and study participants

This survey study was performed in a 595-bed academic teaching hospital from September 1st to November 30th 2015. All allied health care professionals with direct patient contact were asked to participate in the study. Of the 185 allied healthcare workers 117 (63.2%) were registered nurses, 10 (5.4%) nursing auxiliaries, 55 (29.7%) nursing students, and 3 (1.6%) did not define their tatus. The survey was offered to all inpatients admitted to wards of the departments of orthopedics, urology, and general medicine during the study period. Furthermore, study participants were recruited from patients attending an orthopedic, a neurosurgical, and a rheumatologic outpatient clinic of the teaching hospital during the study period. Recruitment of patients was independent of a previous history or present risk of receiving a blood transfusion.

### Survey design

A modified version of a survey described by Vetter et al. was used for this study [8]. The survey consisted of an opening question on the overall risk perception in terms of administration of blood transfusions followed by questions on the degree of concern about five known side effects of blood transfusions. The surveyed adverse effects included allergic reactions, fever, dyspnea, infections with HIV/AIDS or hepatitis C virus (HCV), and donor-recipient-incompatible blood transfusions followed by perceptions on the occurrence rate of these five adverse effects and the information sources these perceptions were based on (primary care physician, family and friends, the media, internet). A 5-point Likert-scale risk score (1 = no concern/no occurrence, 2 = a little concerned/occurs rarely, 3 = moderately concerned/occurs sometimes, 4 = often concerned/occurs frequently, 5 = very often concerned/occurs very frequently) was used for responses. In addition, demographic data including age, sex, marital status, area of living and whether the patient/health care professional has been a blood donor was recorded. Health care professionals were also asked to name the medical specialty they were currently working in. A translated version of the paper survey is available in the Additional file 1.

### Statistical analysis

Results of continuous data are expressed as median with interquartile rage (IQR). Categorical data are presented as frequencies (%) with 95% confidence intervals (CI). Scores for risk perception and perception of incidences were collapsed into two dependent outcome categories of risk scores ranging from 1 to 3 and from 4 to 5. Differences between groups were tested by the non-parametric (exact) Wilcoxon-Mann-Whitney test for independent groups. Frequencies were tested by the (exact) Chi-square-test in contingency tables. Binary logistic regression modeling was conducted to evaluate associations between socio-demographic variables and the assessment of transfusion risks. All variables available were introduced in the models. Regression modeling was conducted using R (http://www.r-project.org), all other calculations were performed with *Predictive Analytics SoftWare* (PASW), Version 22. A two-tailed *p*-value < 0.05 was considered statistically significant.

## Results

### Demographics of patients and caregivers

Of 551 recruited patients and 202 recruited caregivers, 506 patients (91.8%) and 185 caregivers (91.6%) completed the survey (91.8% response rate). Demographic data of both groups is shown in Table 1. Compared to patients, caregivers were younger, more often female, and less often married (Table 1). More than half of the caregivers were blood donors (51.4%) compared to 24.9% of patients (*P* < 0.001).

**Table 1:** Demographics of Caregivers and Patients

	Caregivers		Patients		*P*
	*n* = 185		*n* = 506		
Age, years, median (IQR)	31	(23–47)	51	(36–61)	< 0.001
Gender, male, n (%)	50	(27.0)	266	(52.6)	< 0.001
Marital status, n, (%)					< 0.001
Single	95	(51.4)	135	(26.9)	
Married	79	(42.7)	304	(60.1)	
Divorced	9	(4.9)	37	(7.3)	
Widowed	2	(1.1)	29	(5.7)	
Residential location, n (%)					0.073
Urban	46	(24.9)	82	(16.2)	
Suburban	44	(23.8)	132	(26.1)	
Municipal	39	(21.1)	128	(25.3)	
Rural	56	(30.3)	164	(32.4)	
Blood donor, n (%)	95	(51.4)	126	(24.9)	< 0.001
Denial of blood transfusion, n (%)	2	(1.1)	22	(4.3)	0.036

### Perception of overall transfusion-associated risk

Blood transfusions were considered to be generally harmful by 55 patients (10.9%) and 27 caregivers (14.6%, *P* = 0.180). Categorical rejection of a transfusion of allogenic blood occurred more frequently in patients (patients: 4.3% vs. caregivers: 1.1%, *P* = 0.036).

### Comparison of risk perception and perceived incidences of transfusion-associated risks between patients and caregivers

**Fig. 1:**
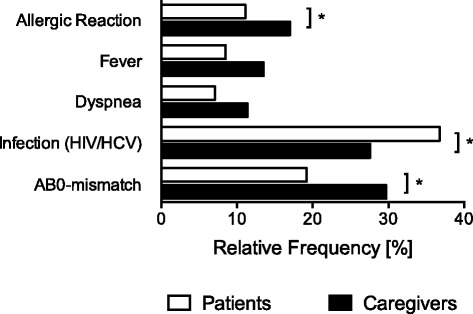
Comparison of risk perception for five specific transfusion-associated risks between patients and caregivers. Relative frequencies for risk perception of allergic reactions, fever, dyspnoea, infections with human immunodeficiency virus (HIV)/hepatitis c virus (HCV), or AB0-mismatch transfusions associated with blood transfusions in patients (n = 506) and caregivers (n = 185); *P < 0.05

**Fig. 2:**
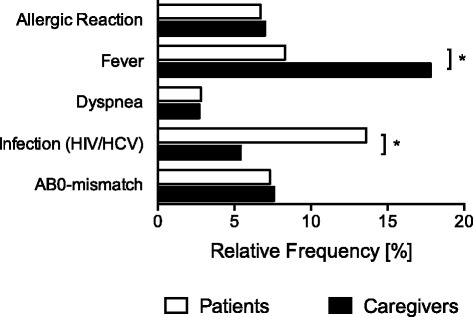
Comparison of perceived incidences for five specific transfusion-associated risks between patients and caregivers. Relative frequencies for perceived incidences of allergic reactions, fever, dyspnoea, infections with human immunodeficiency virus (HIV)/hepatitis c virus (HCV), or AB0-mismatch transfusions associated with blood transfusions in patients (*n* = 506) and caregivers (*n* = 185); *P < 0.05

To study risk perception of blood transfusions in patients and caregivers, participants of this study were asked to indicate their level of concern for five classical blood transfusion-associated adverse effects. In addition, participants were asked to estimate the incidences of these five transfusion-associated effects. Patients and caregivers were often and very often concerned of transfusion-associated transmissions of infections such as HIV or HCV (patients: 36.8% vs. caregivers: 27.6%, *P* = 0.024; Fig. 1). Caregivers ranked the risks of receiving an AB0-mismatch transfusion or an allergic reaction after blood transfusion significantly higher than patients (AB0-mismatch: patients: 19.2% vs. caregivers: 29.7%, *P* = 0.003; allergic reaction: patients: 11.1% vs. caregivers: 17.3%, *P* = 0.030; Fig. 1). Transmissions of infections were perceived to be a frequent and very frequent complication by 13.6% of the patients compared to 5.4% of the caregivers (P = 0.003; Fig. 2). In contrast, 8.3% of the patients compared to 17.8% of the caregivers estimated that transfusion-associated fever is a common and very common adverse effect of blood transfusions (*P* < 0.001; Fig. 2). Allergic reactions, dyspnea, and AB0-mismatch transfusions were estimated to have a high or very high incidence by less than 8% of all participants with no different perceptions of incidences between patients and caregivers (Fig. 2).

Taken together, these results demonstrate that patients were mostly concerned of infection-transmissions associated with blood transfusion and rank the incidences of this complication highest. In contrast, caregivers were aware of the high incidences of transfusion-associated febrile reactions, the low risks of infection-transmissions, and were mostly worried of fatal AB0-mismatch complications associated with the transfusion of allogenic blood products.

### Comparison of risk perception and perceived incidences of transfusion-associated risks between in- and outpatients

In order to test whether risk perception and estimated incidences for adverse effects associated with blood transfusions differed between hospitalized patients and outpatients, the group of participating patients was divided into these two groups. Table 2 indicates that the general perception of blood transfusion as a health hazard and the levels of concern for the five predefined blood transfusion-associated adverse effects did not significantly differ between in- and outpatients in our study population. Furthermore, estimations of incidences of transfusion-associated adverse effects were similar for inpatients and outpatients (Table 2).

**Table 2:** Comparison of risk perception and perceived incidences of transfusion-associated risks between in- and outpatients

	Patients				
	Hospitalized		Outpatients		*P*
	*n* = 284		*n* = 222		
Risk Perception, n, (%)					
Risk in general	30	(10.6)	25	(11.3)	0.802
Allergic Reaction	30	(10.6)	26	(11.7)	0.683
Fever	24	(8.5)	19	(8.6)	0.966
Dyspnea	26	(9.2)	10	(4.5)	0.054
Infection (HIV/HCV)	108	(38.0)	78	(35.1)	0.503
ABO-mismatch Transfusion	60	(21.1)	37	(16.7)	0.206
Perceived Incidence, n, (%)					
Allergic Reaction	17	(6.0)	17	(7.7)	0.456
Fever	27	(9.5)	15	(6.8)	0.266
Dyspnea	8	(2.8)	6	(2.7)	1.000
Infection (HIV/HCV)	39	(13.7)	30	(13.5)	0.943
ABO-mismatch Transfusion	26	(9.2)	11	(5.0)	0.072

### Comparison of information sources used by patients and caregivers

**Fig. 3:**
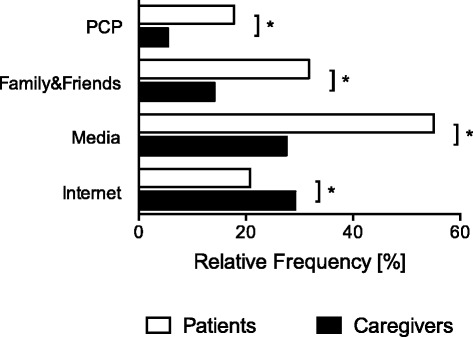
Comparison of information sources used by patients and caregivers. Information sources such as primary care physicians (PCP), family and friends, the media, or the Internet that were considered relevant by patients (n = 506) and caregivers (n = 185) to influence the own opinion about blood transfusions; *P < 0.05

For evaluation of information sources that influenced the risk perception of the study participants about blood transfusions, patients and caregivers were asked whether information provided by their primary care physicians, family and friends, the media, or the Internet were relevant for their opinion about blood transfusions. Compared to caregivers, patients’ knowledge about blood transfusions was more often influenced by primary care physicians, family and friends, and media like radio, television, or newspapers (Fig. 3). Besides their professional education, caregivers used the Internet more often than patients to obtain information about blood transfusions (Fig. 3).

### Association of sociodemographic factors with the perceived risks of blood transfusion in patients and caregivers

A binary logistic regression model was used to estimate the association of sociodemographic factors with the perceived risk associated with blood transfusions in general and with regard to the five specific blood transfusion-associated adverse effects (allergic reaction, fever, dyspnea, infection-transmission, and AB0-mismatch transfusion). Categorical denial of blood transfusions was associated with an overall increased risk perception of blood transfusions among patients (Table 3). In contrast, none of the demographic factors was associated with perception of an increased risk associated with blood transfusions among caregivers.

**Table 3:** Binary logistic regression models estimating the association of sociodemographic factors with overall risk assessment of blood transfusion in all study participants as well as in the subgroups of patients and caregivers

	All participants (*n* = 691)		Patients (n = 506)		Caregivers (n = 185)	
	OR (95%CI)	*P*	OR (95%CI)	*P*	OR (95%CI)	*P*
Age, years	1.00 (0.97–1.02)	0.62	1.01 (0.98–1.03)	0.61	1.02 (0.97–1.07)	0.36
Sex, female	1.57 (0.96–2.62)	0.08	1.67 (0.92–3.06)	0.09	1.06 (0.40–3.04)	0.91
Marital status^a^						
Unmarried	1.21 (0.63–2.31)	0.57	1.36 (0.60–2.97)	0.46	1.32 (0.25–4.89)	0.67
Divorced	1.64 (0.68–3.62)	0.24	2.26 (0.85–5.52)	0.09	0.58 (0.03–3.79)	0.63
Widowed	0.23 (0.01–1.19)	0.16	0.26 (0.01–1.39)	0.21	n/a	0.99
Residence^b^						
Suburban	1.16 (0.60–2.29)	0.67	1.35 (0.59–3.26)	0.48	0.79 (0.23–2.59)	0.70
Municipal	0.76 (0.37–1.58)	0.46	1.07 (0.44–2.70)	0.88	0.34 (0.07–1.34)	0.15
Rural	0.62 (0.30–1.25)	0.18	0.55 (0.21–1.44)	0.22	0.76 (0.25–2.29)	0.61
Blood donor	1.57 (0.94–2.61)	0.08	1.26 (0.64–2.42)	0.49	2.13 (0.88–5.51)	0.10
Denial of transfusions	5.53 (2.15–13.6)	< 0.001	5.46 (1.97–14.5)	< 0.001	9.74 (0.34–27.9)	0.13

^a^compared to married subjects; ^b^compared to subjects residing in a city

Results of five binary logistic regression models estimating the association of sociodemographic factors with the assessment of five specific risks of blood transfusions suggest that in patients but not in caregivers, consequent denial of blood transfusions, female sex and martial status are associated with increased fear of transfusion-associated adverse effects (Table 4).

**Table 4:** Five binary logistic regression models estimating the association of sociodemographic factors with the assessment of five specific risks of blood transfusion in (A) patients (n=506) and (B) caregivers (n=185)

	Allergic Reaction		Fever		Dyspnea		Infection		AB0-mismatch	
	OR (95%CI)	P	OR (95%CI)	P	OR (95%CI)	P	OR (95%CI)	P	OR (95%CI)	P
A										
Age, years	1.00 (0.98–1.02)	0.98	0.99 (0.97–1.02)	0.57	1.00 (0.98–1.02)	0.87	1.00 (0.99–1.02)	0.70	0.98 (0.97–1.00)	0.07
Sex, female	1.85 (1.03–3.41)	0.04	2.85 (1.43–6.01)	<0.01	1.66 (0.80–3.51)	0.18	1.42 (0.97–2.10)	0.07	1.35 (0.85–2.14)	0.20
Marital status^a^										
Unmarried	0.80 (0.35–1.74)	0.58	0.97 (0.39–2.34)	0.95	1.63 (0.64–4.07)	0.30	1.65 (0.98–2.78)	0.06	0.81 (0.43–1.52)	0.52
Divorced	0.51 (0.11–1.66)	0.32	0.70 (0.15–2.38)	0.61	0.33 (0.02–1.85)	0.30	2.21 (1.07–4.60)	0.03	1.50 (0.64–3.28)	0.33
Widowed	0.20 (0.01–1.05)	0.13	1.18 (0.25–4.04)	0.81	1.04 (0.15–4.25)	0.96	1.45 (0.63–3.31)	0.38	1.12 (0.35–3.04)	0.83
Residence^b^										
Suburban	0.85 (0.35–2.12)	0.72	1.10 (0.41–3.12)	0.85	0.75 (0.24–2.38)	0.62	1.17 (0.65–2.12)	0.60	1.32 (0.65–2.74)	0.45
Municipal	0.89 (0.36–2.24)	0.80	0.73 (0.25–2.22)	0.58	1.06 (0.37–3.23)	0.92	0.65 (0.35–1.20)	0.16	1.11 (0.54–2.36)	0.78
Rural	0.83 (0.35–2.04)	0.67	1.09 (0.42–3.04)	0.86	0.97 (0.35–2.91)	0.96	0.97 (0.55–1.72)	0.90	1.10 (0.55–2.25)	0.80
Blood donor	0.61 (0.27–1.26)	0.21	0.91 (0.39–1.93)	0.81	0.99 (0.40–2.22)	0.98	0.51 (0.31–0.81)	<0.01	1.02 (0.60–1.71)	0.93
Denial of transfusions	6.88 (2.57–18.0)	<0.001	7.39 (2.49–20.7)	<0.001	8.20 (2.77–22.9)	<0.001	4.74 (1.85–13.8)	<0.01	2.60 (1.01–6.52)	<0.05
B										
Age, years	1.00 (0.94–1.05)	0.95	0.99 (0.92–1.05)	0.67	1.05 (0.99–1.11)	0.09	1.03 (0.99–1.07)	0.12	0.99 (0.95–1.03)	0.76
Sex, female	0.78 (0.30–2.11)	0.60	0.55 (0.20–1.65)	0.27	0.94 (0.31–3.20)	0.91	1.32 (0.60–3.10)	0.50	1.32 (0.60–3.04)	0.50
Marital status^a^										
Unmarried	2.55 (0.71–1.05)	0.16	3.35 (0.74–17.7)	0.13	8.31 (1.73–48.9)	0.01	2.38 (1.01–8.37)	0.05	1.74 (0.64–4.86)	0.28
Divorced	2.26 (0.29–1.20)	0.37	4.87 (0.59–3.01)	0.10	1.55 (0.07–1.19)	0.71	0.73 (0.09–3.45)	0.71	0.92 (0.13–4.30)	0.92
Widowed	n/a	0.99	n/a	0.99	n/a	0.99	n/a	0.99	n/a	0.99
Residence^b^										
Suburban	4.37 (0.11–1.49)	0.21	1.00 (0.22–4.35)	0.99	8.14 (0.17–3.53)	0.78	0.64 (0.21–1.86)	0.42	1.10 (0.40–3.02)	0.85
Municipal	0.60 (0.17–1.96)	0.41	1.56 (0.38–6.35)	0.53	1.21 (0.29–4.98)	0.79	1.93 (0.73–5.19)	0.18	1.81 (0.69–4.82)	0.23
Rural	1.05 (0.38–2.94)	0.92	2.66 (0.82–9.81)	0.12	1.38 (0.38–5.30)	0.62	1.32 (0.53–3.39)	0.55	1.21 (0.49–3.04)	0.68
Blood donor	0.87 (0.51–1.45)	0.42	1.51 (0.61–3.92)	0.38	2.33 (0.86–6.91)	0.11	1.32 (0.67–2.67)	0.42	1.38 (0.70–2.71)	0.35
Denial of transfusions	n/a	0.99	5.53 (0.19–169)	0.27	n/a	0.99	3.26 (0.12–90.1)	0.42	2.06 (0.07–5.52)	0.62

^a^compared to married subjects; ^b^compared to subjects residing in a city

## Discussion

A significant part of interviewees perceived transfusion of allogenic blood as a general health hazard. Patients, irrespective whether they were in- or outpatients, were mostly worried about infection-transmissions and believed this is the most frequent transfusion-associated complication. Consequent denial of blood transfusions and female sex were associated with increased fear of transfusion-associated adverse effects in patients. Caregivers were mainly concerned of AB0-mismatch transfusions and based on their professional education were aware of the high frequency of transfusion-associated febrile reactions.

Despite an increased safety profile and very low complication rates of blood transfusions in recent decades, a respectable number of patients (10.9%) and caregivers (14.6%) were concerned about blood transfusions. These findings are concordant with responses by patients in the United States [8]. Moreover, Vetter and colleagues demonstrated that anesthesiologists and surgeons ranked the overall risk of blood transfusions significantly higher compared to their patients, but the physicians perceived the risks of five specific transfusion-associated adverse effects lower than their patients [8]. In the current study, the specific risk of infection-transmissions with blood transfusions were perceived lower by the caregivers than by their patients while risks of allergic reactions and AB0-mismatch transfusions were perceived higher.

Risk perception in general is mainly driven by intuition instead of probability-based assessments [2, 9]. A risk is perceived higher the less control a person may have over the given risk. Risk perception about blood transfusions is dependent on a balance of a person’s knowledge about the risks of blood transfusions and the degree of confidence that this knowledge is correct and sufficient [10]. As expected, medical experts like general practitioners and anesthesiologists, demonstrate deeper knowledge and higher confidence in the knowledge about blood transfusions than the general public [10, 11]. Infection-transmissions caused by blood transfusions are low with currently only one transmission in more than 4 million transfusions for HIV and in more than 10 million transfusions for HCV [12]. Based on their professional exposure to blood transfusions, allied caregivers might be aware of the low incidence of infection-transmissions of HIV and HCV with blood transfusions [13]. However, their apprehension of infection-transmissions with blood transfusion is higher compared to the apprehension that was quantified for physicians in previous surveys [8, 10, 11]. Contrary to the view in public, the leading causes of transfusion-related deaths are TRALI and hemolytic transfusion reactions, many of the latter based on AB0-mismatch transfusions [5]. In Germany, lethal AB0-mismatch transfusions occur in about one of a million transfusions [14]. In contrast to patients, allied caregivers rank this fatal complication highest in their risk perception of blood transfusions. Based on their professional education, medical experts might have an understanding of the fatal consequences of an AB0-incompatible transfusion although they seem to be aware of the low incidence of its occurrence. In contrast, patients are mainly dependent on health and medical information by the media and their primary care physicians [15]. The extensive media coverage of transfusion-transmitted HIV-infections in hemophilic patients in the 1980’s formed the public’s fear about infection-transmissions associated with blood transfusions that persist up to the present day [2]. The media was also the dominating information source for patients questioned in this study indicating that this information source can also be used as a powerful tool for education and for transmission of information about the scientific evidence on risks and side effects of blood transfusions.

Sociodemographic factors including older age, female sex, low income and education, married status, and minority background have been described to be associated with increased risk perception of allogenic blood transfusion [16–18]. Accordingly, risk perception of blood transfusions was higher in female patients compared with male patients in this survey. In allied clinical professionals, female sex was not associated with an increased dread of blood transfusions although this group consisted predominantly of women. Older age did not show a heightened risk perception in the patient or caregiver group of this study. However, the average age in both studied groups with 31 years for caregivers and 51 years in the patients groups was low compared to previous studies [8].

Limitations of the present study include its single-center design and a skewness to predominantly female respondents in the caregiver group. Gender is known to have an impact on a persons general risk perception. General risk perception of the interviewee was not assessed. Risk tolerance for comparative risks and other health hazards can have an impact on risk perception about blood transfusions [13]. Furthermore, patients were recruited from medical and surgical specialties with low and medium transfusion incidences. No patient recruitments were conducted in specialties with high transfusion requirements such as cardiothoracic and vascular surgery, and large visceral, liver, and gynecological surgery. In addition, this survey was designed to assess only risk perception of blood transfusions and reasons for hospitalization were not recorded. Consent for a medical intervention involves risks, benefits and other alternatives. Risk perception about blood transfusions might be overestimated when not balanced against transfusion-associated benefits.

## Conclusions

In conclusion, in the current study we demonstrate that a significant part of patients and caregivers perceive transfusion of allogenic blood as a general health hazard. Patients perceive infection-transmissions as the most frequent and greatest health risk associated with blood transfusions. In contrast, based on their professional education, the risk perception on blood transfusions of caregivers is mainly focused on fatal AB0-mismatch transfusions. Consideration of the patients’ main concerns about blood transfusions and detailed knowledge about the actual incidences and risks of transfusion-associated adverse effects might improve the process of shared decision making when obtaining informed consent before medical procedures and interventions.

## Additional file

Additional file 1: Questionnaire. (PDF 55 kb)
