# Supplementary material for: Risk perception of blood transfusions – a comparison of patients and allied healthcare professionals
Source: BMC Health Serv Res. 2018 Feb 17;18:122. doi: 10.1186/s12913-018-2928-x (PMC5816539; doi:10.1186/s12913-018-2928-x)
Supplement: Supplementary file 1 — Questionnaire. (PDF 55 kb) [file 12913_2018_2928_MOESM1_ESM.pdf]

Additional file 1 (Questionnaire)

1) Do you perceive blood transfusions as a health hazard?

Not at all 1 - 2 - 3 - 4 - 5 very much

2) Are you concerned that the following complications can occur after a blood transfusion?

Allergic reaction
No concern 1 - 2 - 3 - 4 - 5 very often concerned

Fever
No concern 1 - 2 - 3 - 4 - 5 very often concerned

Shortness of breath
No concern 1 - 2 - 3 - 4 - 5 very often concerned

Infection with HIV/hepatitis c virus
No concern 1 - 2 - 3 - 4 - 5 very often concerned

Medical error (like AB0-incompatible blood transfusion)
No concern 1 - 2 - 3 - 4 - 5 very often concerned

3) Please make an estimate guess how often the following complications associated with blood transfusions occur

Allergic reaction
No occurrence 1 - 2 - 3 - 4 - 5 occurs very frequently

Fever
No occurrence 1 - 2 - 3 - 4 - 5 occurs very frequently

Shortness of breath
No occurrence 1 - 2 - 3 - 4 - 5 occurs very frequently

Infection with HIV/hepatitis c virus
No occurrence 1 - 2 - 3 - 4 - 5 occurs very frequently

Medical error (like AB0-incompatible blood transfusion)
No occurrence 1 - 2 - 3 - 4 - 5 occurs very frequently

4) Source of knowledge/opinion about blood transfusions (Please mark)

- General Practitioner
- Family & Friends
- Print media & television
- Internet
- Others

5) Your age: sex:

6) Marital status?
- Married
- Unmarried
- Divorced
- Widowed

7) Residence?
- Urban (City)
- Suburban (Edge of town)
- Municipal
- Rural (Village)

8) Blood donor: Yes/No?

9) Categorical denial of transfusion: Yes/No?
